# The Assessment of the Combined Treatment of 5-ALA Mediated Photodynamic Therapy and Thalidomide on 4T1 Breast Carcinoma and 2H11 Endothelial Cell Line

**DOI:** 10.3390/molecules25215184

**Published:** 2020-11-07

**Authors:** Krzysztof Zduniak, Katarzyna Gdesz-Birula, Marta Woźniak, Kamila Duś-Szachniewicz, Piotr Ziółkowski

**Affiliations:** Department of Pathology, Wrocław Medical University, Marcinkowskiego 1, 50-368 Wrocław, Poland; krzysztof.zduniak@umed.wroc.pl (K.Z.); katarzyna.gdesz@gmail.com (K.G.-B.); kamila.dus-szachniewicz@umed.wroc.pl (K.D.-S.); piotr.ziolkowski@umed.wroc.pl (P.Z.)

**Keywords:** photodynamic therapy, thalidomide, breast carcinoma, in vitro, VEGF

## Abstract

Photodynamic therapy (PDT) is a low-invasive method of treatment of various diseases, mainly neoplastic conditions. PDT has been experimentally combined with multiple treatment methods. In this study, we tested a combination of 5-aminolevulinic acid (5-ALA) mediated PDT with thalidomide (TMD), which is a drug presently used in the treatment of plasma cell myeloma. TMD and PDT share similar modes of action in neoplastic conditions. Using 4T1 murine breast carcinoma and 2H11 murine endothelial cells lines as an experimental tumor model, we tested 5-ALA-PDT and TMD combination in terms of cytotoxicity, apoptosis, Vascular Endothelial Growth Factor (VEGF) expression, and, in 2H11 cells, migration capabilities by wound healing assay. We have found an enhancement of cytotoxicity in 4T1 cells, whereas, in normal 2H11 cells, this effect was not statistically significant. The addition of TMD decreased the production of VEGF after PDT in 2H11 cell line. Our results reveal enhanced effectiveness of 5-ALA-PDT with TMD treatment compared to 5-ALA-PDT or TMD treatment alone. The addition of TMD may be a promising proceeding of the anti-tumor effect of PDT by decreasing the VEGF concentration in the culture medium. Further studies, including testing on different cell lines, are needed to confirm this assumption.

## 1. Introduction

Photodynamic therapy (PDT) is a minimally invasive procedure of treatment of different diseases, mainly cancers [1]. Due to the irradiation of treated tissue with light at the proper wavelength, previously pre-excited by a delivered photosensitizing agent, it generates reactive oxygen species (ROS) [1]. 5-aminolevulinic acid (5-ALA)-PDT is based on exogenous administration of prodrug—5 aminolevulinic acid (5-ALA). After 4 h of incubation, increased production of protoporphyrin IX (PpIX) in tumor cells mitochondria is observed. Accumulated PpIX act as a photosensitizer in targeted cells. After red light irradiation, excitation of the photosensitizer results in the formation of reactive oxygen species that exerts cytotoxic effects [2]. In the neoplastic process ROS generation results in the deterioration of cancerous tissue via several different manners [1]. The most apparent way is the induction of tumor cell apoptosis, autophagy and necrosis due to the damaging of external and internal lipid membranes and the release of caspase activators [1]. PDT also influences vascular-endothelial growth factor (VEGF) expression and directly damages vessel wall, which results in the reduction of tumor vasculature and blood supply to tumor cells [3]. As a result of tissue injury, cells of acute inflammatory response infiltrate PDT-treated tissue [1]. Inflammatory infiltrate consists mostly of neutrophils and macrophages; however, CD8-positive T-cells are also represented in large numbers after PDT [1].

Several authors have made an effort to enhance PDT anti-tumor effectiveness. Many researchers worked on novel photosensitizing agents [4], light sources [5], and also novel ways of photosensitizer targeting [6]. There are also attempts to deliver photosensitizers to tumor cells more effectively using local hyperthermia or creating liposomal forms of classic photosensitizers [7]. Moreover, PDT has been experimentally combined with various treatment methods, like radiotherapy [8]. Correlation between PDT and chemotherapeutic agents or natural plant derivatives to improve the effectiveness of combined therapy has also been tested [9,10,11].

The study of breast cancer microenvironment and angiogenesis resulted in the research of VEGF expression. It is assumed that VEGF expression in normal glandular structures is lower than in breast lesions [12]. Thus, the modulation of VEGF production might have therapeutic effects in cancer angiogenesis. One of the most famous inhibitors of angiogenesis currently used in clinical practice is thalidomide (TMD). Today, this drug is successfully used in the treatment of plasma cell myeloma [13] and is also under study for breast cancer treatment [14]. Besides its bad reputation in the past decades because of teratogenic effects in the first trimester of fetus development—it has been used in pregnant women in the 20th century as a sedative and antiemetic agent [15]—thalidomide antiproliferative and anti-tumor effects are astonishing and related to several different modes of action [16]. It has been shown that thalidomide has proapoptotic activity due to inhibition of NFkB signaling and induction of caspase activity [16]. Subsequently, it shows antiangiogenic activity due to decreasing of Tumor Necrosis Factor α (TNFα) and VEGF expression [16]. It has also been found that thalidomide induces oxidative stress in cell lines [16]. Therefore, properties of thalidomide are, to some extent, similar to PDT-induced effects. 

Our study aimed to investigate whether thalidomide enhances 5-ALA-mediated photodynamic therapy in murine 4T1 breast cancer and 2H11 endothelial cell lines.

## 2. Results

### 2.1. MTT Assay

**Figure 1:**
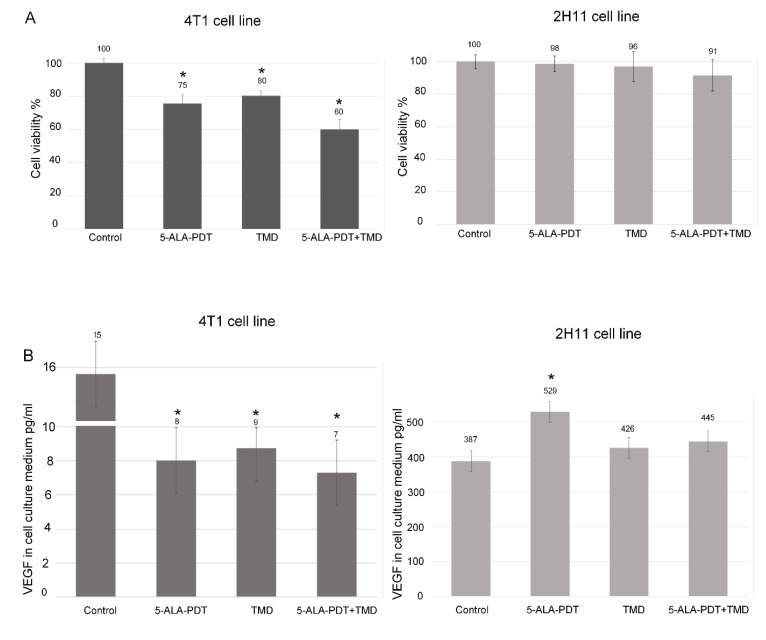
(**A**) Results of MTT assay on 4T1 and 2H11 cells after 5-aminolevulinic acid (5-ALA)-photodynamic therapy (PDT), thalidomide-5-ALA-PDT, and in control groups. Results are presented as means ± standard deviations (*n* = 3). * indicates *p* < 0.01. (**B**) Vascular Endothelial Growth Factor (VEGF) concentration (in pg/mL) in 4T1 and 2H11 cells in the culture medium. Results are presented as means ± standard deviations from two independent experiments. * indicates *p* < 0.01.

To evaluate cytotoxic and phototoxic effect of the described treatment, both 4T1 and 2H11 cell lines were tested using MTT assay. An enhanced cytotoxic and phototoxic effect of 5-ALA-PDT and thalidomide treatment was observed. In 4T1 cell line, in comparison to non-treated control samples (100% viability), 5-ALA-PDT-treated cells mean viability after 48 h was 75%, in TMD-treated sample viability rate was 80%, and in combined 5-ALA-PDT + TMD-treated samples, viability was 60%. The differences between control and treated samples were statistically significant. On the other hand, the same dose of 5-ALA-PDT and thalidomide used for 2H11 cells presented very low phototoxicity and cytotoxicity, respectively. The viability between control and treated samples did not exceed statistical significance. 5-ALA-PDT-treated samples mean viability was 98%, in TMD-treated samples it was 96%, and in 5-ALA-PDT- and TMD-treated samples viability amounted to 91%. Control groups for 5-ALA, light only and control of 0.5% of DMSO in both cell lines showed a viability rate of almost 100% (See Supplementary Data). The results of the MTT assay are shown in Figure 1A.

### 2.2. Apoptosis Assay

**Figure 2:**
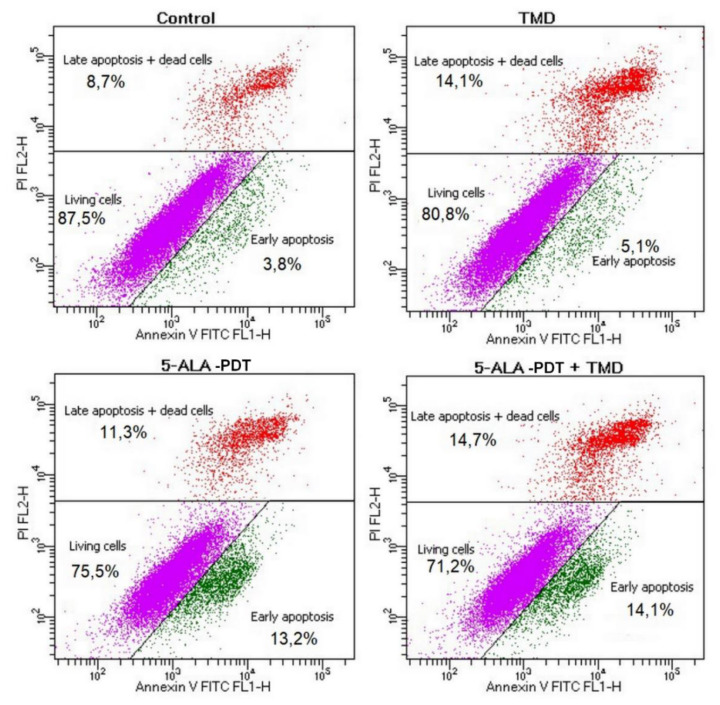
Results present representative images of flow cytometry analysis of viable, early, late apoptotic, and dead 4T1 cells after incubation with 20 µg/mlthalidomide for 48 h, 3 mM concentration of 5-ALA-PDT (5 J/cm^2^), -5-ALA-PDT+ thalidomide for 48 h treatment in comparison to the control group without any treatment. Annexin V-FITC and PI Apoptosis Detection Kit (Abcam). All samples were analyzed using FACS Calibur flow cytometer (Beckton Dickinson, NJ, USA). All samples were measured independently in triplicate.

In order to assess the apoptotic and necrotic 4T1 cells after the proposed treatment flow cytometry analysis using Annexin-5 and propidium iodide was performed. We observed an increase of early and late apoptosis after the combination of 5-ALA-PDT with TDM (almost 30% of cells) treatment in our mouse breast cancer model as compared to control cells. Cells treated only with 5-ALA-PDT or TMD alone underwent programmed cell death in 25% and 20%, respectively. The percentage of living cells, early apoptotic, and late apoptotic with dead cells results are presented in Figure 2.

### 2.3. ELISA Assay 

The next experiment was performed to assess whether and to what extent VEGF production in both cell lines was disrupted by the treatment. The VEGF concentration in cell culture media in 2H11 and 4T1 cell lines was measured by the ELISA test. We have found that 5-ALA mediated PDT significantly increased the concentration of VEGF (529 pg/mL) in the culture medium of 2H11 cells in comparison with the control group—387 pg/mL. Whereas, in the sample treated only with thalidomide and 5-ALA-PDT with TMD, the concentration of VEGF in the culture medium was slightly increased to 426 and 445 pg/mL, respectively. In 4T1 cells, the VEGF level was reduced by half in treated samples compared to the control group. In control samples, VEGF production was at almost 16 pg/mL, while, in 5-ALA-PDT, TMD, and 5-ALA-PDT + TMD, it was at 8 pg/mL, 9 pg/mL, and 7 pg/mL, respectively. The results are presented in Figure 1B.

### 2.4. Wound Healing Assay 

**Figure 3:**
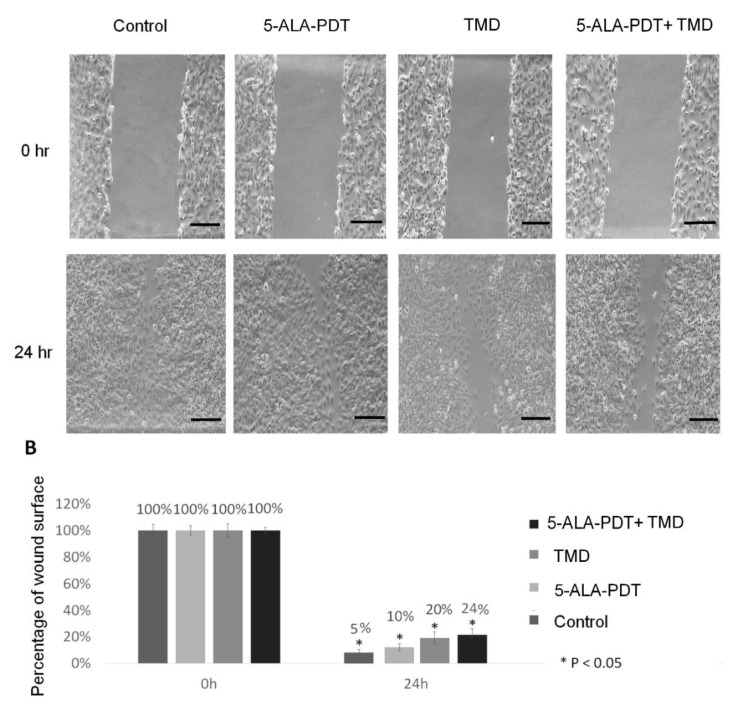
Wound-healing assay in time point 0 and 24 h 2H11 endothelial cell line. (**A**) Representative images show that after 24 h, the scrap in control cells and treated with 3 mM concentration 5-ALA-PDT is minimal compared to samples incubated with 20 µg/mL thalidomide for 48 h alone and in combination with 3 mM concentration 5-ALA-PDT, where there is limited migration of cells observed. (**B**) Quantification of cell migration for 2H11 cells. Results are presented as the percentage of the wound surface. The initial wound area is expressed as 100% at 0 h. The experiment was performed in triplicate. Scale bars represent means with standard deviation. Treatment vs. control: * *p* < 0.05. Scale bar = 100 µm.

To evaluate whether 5-ALA-PDT and TMD decreases 2H11 cell motility, the wound healing test was performed. The results show that the migration abilities of 2H11 endothelial cells were attenuated by thalidomide alone in 20%, but, in combination with 5-ALA-PDT, this effect of motility inhibition after 24 h was increased to 24%. Cells treated only with PDT (5-ALA + irradiation) showed similar migration capabilities compared to control cells. The wound in these samples closed after 24 h of incubation almost completely. The results are presented in Figure 3.

## 3. Discussion

A large number of research groups have studied a combination of photodynamic therapy with various anti-cancer treatment methods [1,17]. Anand et al. reported that 5-fluorouracil pretreatment of murine squamous cell carcinoma and implanted A431 and 4T1 tumors resulted in enhancement of 5-ALA mediated PDT, due to increased protoporphyrin IX levels in cancerous cells [18]. Another study by Ali et al. focused on PDT enhancement using methotrexate and doxorubicin. The authors have found synergistic interaction between PDT and chemotherapeutic agents, which most likely results from the more effective mitochondrial accumulation of photosensitizer [19]. Another group has studied the influence of artemisinin on PDT effectiveness in the mouse breast cancer model. The effects of combined therapy also enhanced 5-ALA-PDT therapy [17].

According to several well-documented similarities in the mode of action of PDT and thalidomide, we observe significant biological effects of such a combination in our experimental model. Our results are consistent with previous findings that VEGF may be initially overexpressed after PDT in treated cells [20,21]. In our model, we demonstrated this effect in endothelial cells, 2H11. There were no differences in VEGF production in 4T1 cells among the thalidomide alone, 5-ALA-PDT, 5-ALA-PDT with TMD groups. Nevertheless, in all of the above-described groups, VEGF expression was around twice lower than in the control group. It has also been found that thalidomide may reduce the expression of VEGF in endothelial and cancer cells [16]. In our study, we found that 5-ALA-PDT and TMD lower VEGF expression levels in 4T1 cells, but this effect was no statistically significant in comparison to 5-ALA-PDT or TMD only treated cells. Interestingly, in 2H11 cells TMD alone does not decrease the VEGF primary expression; however, it decreases after treatment with 5-ALA-PDT. 

Although we observed an impressive influence of thalidomide on VEGF secretion levels after PDT in endothelial cells after 48 h of incubation, this effect was not translated on proliferation activities. The same dose for both cell lines 3 mM of 5-ALA and 5 J/cm^2^ of light did not affect the viability of 2H11 cells. Contrarily, cytotoxicity of 5-ALA-PDT alone or in combination with TMD significantly decreased the viability of 4T1 cells. Basing on MTT and apoptosis test results, we conclude that 2H11 endothelial cell line, in terms of cytotoxicity, is much less susceptible to our treatment protocols than 4T1 tumor cell line. This is an expected result, as PDT gives a much less toxic effect in normal cells [22]. Moreover, we observed a discrepancy in cell viability between MTT assay and flow cytometry. This result might by supported by the fact that MTT assays rely on the metabolic activity determined mainly by the function of mitochondria. The higher the metabolic activity of cells, the higher the positive results in MTT assays. When a cell starts reducing its metabolic activity, changes in MTT assays can be seen. This might happen without any apoptotic processes seen in flow cytometry analysis.

Because of the very high proliferative capabilities of 2H11 cell lines, we investigated the proposed treatment on migration capabilities by wound healing assay. Our results confirm a slight decrease in motility. Control cells and 5-ALA-PDT nearly filled the wound (5% remaining) by 24 h, whereas the wound in TMD- and PDT-treated cells remained unfilled in 30% in comparison to time 0 h. This result might be supported by the impaired expression of VEGF [23], but it needs to be confirmed on another group, where the recombinant murine VEGF should be used to reverse the observed phenotype.

Enhanced effects of the combination of 5-ALA-PDT with TMD in 4T1 cell line may be related to overtly malignant features of the selected tumor cell line, since 4T1 cells have shown very high proliferation capabilities. These cells also have highly anaplastic morphology, strong metastatic potential [24] and have been used as a triple-negative breast carcinoma model [25]. In addition, de Souza et al. observed that combined therapy with thalidomide acts better in suppressing the growth in murine mammary carcinoma 4T1 [14]. Although our results indicate the proposed treatment as a promising strategy in breast cancer treatment, the key aspects of the effectiveness of thalidomide and 5-ALA-PDT treatment require in vivo studies to support the idea. Furthermore, considering that PDT has limited data on resident stromal cells in the contextual microenvironment, it would be essential to analyze other cell types, such as fibroblasts, for example, in mouse 3TS cells in order to VEGF production after proposed treatment.

Our results suggest that the addition of TMD may be a promising proceeding aimed at enhancement of the anti-tumor effect of PDT by decreasing the VEGF concentration in the culture medium. Further studies, including testing on different cell lines with different PDT and TMD doses, are needed to confirm this assumption.

## 4. Materials and Methods 

### 4.1. Cell Lines

4T1 murine breast carcinoma cell line and 2H11 murine endothelial cell line were purchased from American Type Culture Collecion and cultured in RPMI 1640 medium (4T1) and DMEM (2H11) supplemented with 10% fetal bovine serum (FBS), respectively. All cell culture reagents were bought from Gibco, (Thermo Fisher Scientific Inc., Walthman, MA, USA). Cells were incubated in 37 °C and 5% CO_2_ in a humidified atmosphere. For experimental procedures, cells from 5th to 10th passage were used. 

### 4.2. 5-ALA-PDT + Thalidomide (TMD) Therapy Protocol

Cells were incubated with 5-aminolevulinic acid (5-ALA) (Sigma Aldrich, Munich, Germany) dissolved in culture media to achieve 3 mM concentration. After 4 h of incubation, cells were rinsed twice with phosphate buffered saline (PBS). Irradiation was carried out in cell culture media without FBS and phenol red. Cells were irradiated with red light using a halogen lamp (Penta Lamps, Teclas, Lugano, Switzerland). Five joules per centimeter squared of energy was delivered during 83 s exposure with 50 mW/cm^2^ to each sample at the wavelength 630+/−20 nm. Control groups comprised of cells exposed to light only, 5-ALA, or left without any treatment. After cells irradiation, thalidomide (Sigma Aldrich) was added to selected wells. We used 20 mg/mL stock concentration of thalidomide, which was dissolved in DMSO, then the appropriate volume was added to culture media to obtain 20 µg/mL final concentration. DMSO concentration in media did not exceed 0,5% and did not affect the cells. Cells were incubated with thalidomide for 48 h. The second control group of cells comprised those exposed to TMD for 48 h only.

### 4.3. MTT Assay 

The cytotoxicity was measured by the 3-(4,5-dimethylthiazol-2-yl)-2,5-diphenyltetrazolium bromide (MTT) reduction assay (Sigma Aldrich, Germany). 4T1 and 2H11 cells were seeded on 48 well plates in 2 × 10^3^ cells/well. Cells were incubated with a single medium change after the first 24 h. Next, a therapy protocol was carried out. After incubation, cells after 5-ALA-PDT or 5-ALA-PDT with thalidomide, and from control groups were gently rinsed with PBS once, then incubated with MTT solution for the next 4 h. Then the medium was removed and formazan crystals were dissolved in DMSO (Sigma Aldrich, Germany). Light absorbance was measured using a plate reader BioTek ELX800 multi-well reader (BioTek, Winooski, VT, USA) at a wavelength of 570 nm. The absorbance of the samples was counted as the percentage of viable cells (VC) calculated according to the formula: VC (%) = (A of experimental group/A of the control group) × 100%. MTT assay was repeated three times, and Figure 1 presents mean bars with standard deviation.

### 4.4. Apoptosis Assay 

For the evaluation of the apoptotic and necrotic cells after treatment, 4T1 cells were seeded on 6-well plates in 1 × 10^6^/well density and treated as described in the experimental protocol. After 48 h of incubation following 5-ALA-PDT treatment with 20 thalidomide cells were trypsinized, washed in PBS, and centrifuged twice, then resuspended in Binding Buffer and stained using Annexin V-FITC and PI Apoptosis Detection Kit (Abcam). All samples were analyzed using FACS Calibur flow cytometer (Becton Dickinson, Franklin Lakes, NJ, USA). Samples that were single-stained with PI or Annexin-V-FICT were used to compensate for the overlapping PI and Annexin-V-FICT, respectively, emission spectra. After staining in the dark for 15 min at room temperature, cells were again centrifuged, resuspened in Binding Buffer and analyzed by flow cytometry. The results are presented as percentage of living, early, late apoptosis, and dead cells. All the samples were measured independently in triplicate, and Figure 2 shows representative images of experiments. 

### 4.5. ELISA Assay

4T1 and 2H11 cells were seeded on 6-well plates in 2 × 10^6^ cells/well density and treated as described in the experiment protocol. After 5-ALA-PDT treatment, cells were incubated for 48 h in culture media without FBS supplementation with TDM applied to samples. Following incubation cell culture media were collected and centrifuged. The supernatant was harvested, condensed twice, and subjected to tests with ELISA Mouse VEGF ELISA Kit (ab100751, Abcam) according to manufacturer’s instruction. After incubation, wells were washed, and horseradish peroxidase (HRP)-conjugated streptavidin was pipetted to the wells. The wells were washed again, and TMB substrate solution was added to the wells for color development. The Stop Solution changes the color from blue to yellow, and the intensity of the color was measured at 450 nm. The bars in Figure 1B present means from two independent experiments with standard deviation.

### 4.6. Wound Healing Assay 

To evaluate the migration properties of 2H11 cells, the wound healing assay was performed. Cells were seeded on 6-well plates in 1 × 10^6^ density. The medium was changed once after 24 h. As cells formed a confluent monolayer, the PDT procedure was conducted. Using a 200 µl pipette tip, a linear scratch was made in each well. Wells were rinsed with PBS and filled with culture medium and with TMD in selected samples. The wound in the bottom of the plate was assessed in 200× magnification in time point 0 and 24 h until the scratch was closed in the control cell sample. The experiment was repeated three times.

### 4.7. Statistical Analysis

Analysis between the groups was conducted using non-parametric test Kruskal-Wallis for not normal distributed data. *p*-value below 0.05 was considered significant. 

## 5. Conclusion

In this study, we showed that thalidomide could enhance 5-ALA-PDT-induced apoptosis in mouse breast cancer model, 4T1 cell line, but not in mouse endothelial cells 2H11. To the authors’ knowledge, this is the first study to evaluate 5-ALA-PDT with TMD as a new therapeutic strategy to increase the cytotoxicity of 5-ALA mediated PDT against tumor cells. However, more in vivo experiments should be performed to confirm the cytotoxicity, as well as VEGF production, after the proposed treatment. Moreover, further studies are needed to investigate the unknown mechanisms of action in vitro and the difference between normal and tumor cells behavior.

## Supplementary Materials

The following is available, Figure S1: Results of MTT assay on 4T1 (A) and 2H11 (B) cells in control groups: control cells without any treatment, cells treated with 3 mM concentration of 5-aminolevulinic acid, cells irradiated during 83 s exposure with 50 mW/cm^2^ at the wavelength 630+/−20 nm, 0.5% DMSO treated cells. Results are presented as means ± standard deviations (*n* = 3).
