# Supplementary material for: The Assessment of the Combined Treatment of 5-ALA Mediated Photodynamic Therapy and Thalidomide on 4T1 Breast Carcinoma and 2H11 Endothelial Cell Line
Source: Molecules. 2020 Nov 7;25(21):5184. doi: 10.3390/molecules25215184 (PMC7664331; doi:10.3390/molecules25215184)
Supplement: Supplementary file 1 [file molecules-25-05184-s001.pdf]

SUPPLEMENTARY

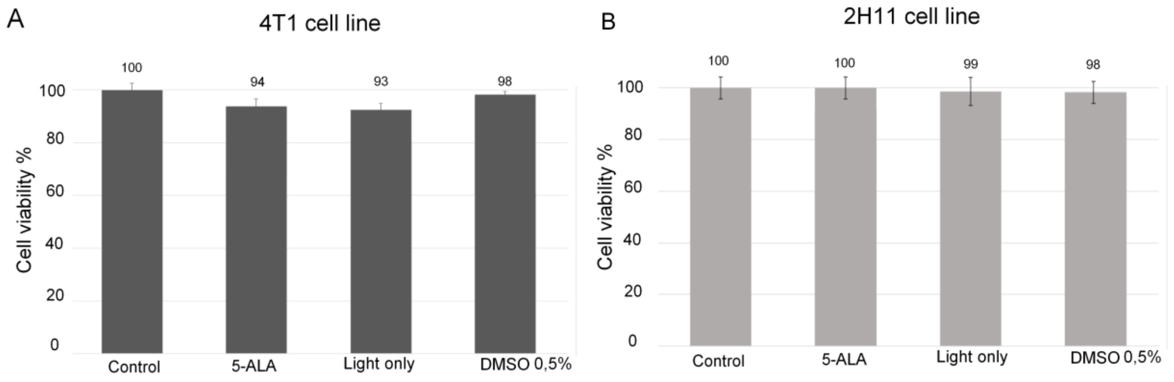

Figure S1. Results of MTT assay on 4T1 (**A**) and 2H11 (**B**) cells in control groups: control cells without any treatment, cells treated with 3 mM concentration of 5-aminolevulinic acid, cells irradiated during 83 s exposure with 50 mW/cm^2 at the wavelength 630+/−20 nm, 0.5% DMSO treated cells. Results are presented as means ± standard deviations (n = 3).
